# Rabies in California

**Published:** 1912-09

**Authors:** 


### ABSTRACTS.

---

Rabies in California.—Dr. Wilbur A. Sawyer, Berkeley, Calif., *Calif. State Jour. of Med.*, Aug., 1912, reports 250 cases of rabies for the year ending Mch. 31, 1912. However, only 2 occurred in man; 240 in dogs; 3 each in cats and cows; 1 each in horse and squirrel.

